# Molecular survey of neglected bacterial pathogens reveals an abundant diversity of species and genotypes in ticks collected from animal hosts across Romania

**DOI:** 10.1186/s13071-018-2756-1

**Published:** 2018-03-20

**Authors:** Martin O. Andersson, Conny Tolf, Paula Tamba, Mircea Stefanache, Gabriel Radbea, Dimitrios Frangoulidis, Herbert Tomaso, Jonas Waldenström, Gerhard Dobler, Lidia Chitimia-Dobler

**Affiliations:** 1 Center for Ecology and Evolution in Microbial Model Systems (EEMiS), Linnaeus University, -391 82 Kalmar, SE Sweden; 2 Institute for Diagnosis and Animal Health, Bucharest, Romania; 3 PAUMI-VET Private Veterinary Clinics, Ilfov County, Snagov, Romania; 4 Sal-Vet Private Veterinary Clinics, Timis County, Timisoara, Romania; 5 Bundeswehr Institute of Microbiology, Neuherbergstrasse 11, D-80937 Munich, Germany; 6 Friedrich-Loeffler-Institut, Institute of Bacterial Infections and Zoonoses, Naumburger Strasse 96a, 07743 Jena, Germany; 7 German Center of Infection Research (DZIF) Partner Munich, Neuherbergstrasse 11, D-80937 Munich, Germany

**Keywords:** Ticks, Neglected bacterial pathogens, Animal hosts, Romania

## Abstract

**Background:**

Ticks are transmitting a wide range of bacterial pathogens that cause substantial morbidity and mortality in domestic animals. The full pathogen burden transmitted by tick vectors is incompletely studied in many geographical areas, and extensive studies are required to fully understand the diversity and distribution of pathogens transmitted by ticks.

**Results:**

We sampled 824 ticks of 11 species collected in 19 counties in Romania. Ticks were collected mainly from dogs, but also from other domestic and wild animals, and were subjected to molecular screening for pathogens. *Rickettsia* spp. was the most commonly detected pathogen, occurring in 10.6% (87/824) of ticks. Several species were detected: *Rickettsia helvetica*, *R. raoultii*, *R. massiliae*, *R. monacensis*, *R. slovaca* and *R. aeschlimannii*. A single occurrence of the zoonotic bacterium *Bartonella vinsonii berkhoffii* was detected in a tick collected from a dog. *Anaplasma phagocytophilum* occurred in four samples, and sequences similar to *Anaplasma marginale/ovis* were abundant in ticks from ruminants. In addition, molecular screening showed that ticks from dogs were carrying an *Ehrlichia* species identical to the HF strain as well as the enigmatic zoonotic pathogen “*Candidatus* Neoehrlichia mikurensis”. An organism similar to *E. chaffeensis* or *E. muris* was detected in an *Ixodes ricinus* collected from a fox.

**Conclusions:**

We describe an abundant diversity of bacterial tick-borne pathogens in ticks collected from animal hosts in Romania, both on the level of species and genotypes/strains within these species. Several findings were novel for Romania, including *Bartonella vinsonii* subsp. *berkhoffii* that causes bacteremia and endocarditis in dogs. “*Candidatus* Neoehrlichia mikurensis” was detected in a tick collected from a dog. Previously, a single case of infection in a dog was diagnosed in Germany. The results warrant further studies on the consequences of tick-borne pathogens in domestic animals in Romania.

**Electronic supplementary material:**

The online version of this article (10.1186/s13071-018-2756-1) contains supplementary material, which is available to authorized users.

## Background

Domestic animals often live in intimate contact with humans, and it has been demonstrated that pet ownership significantly increases the risk of tick encounters for their owners [1]. Thus, dogs and other domestic animals may serve as sentinels for human tick-borne diseases, and, furthermore, might even constitute a potential reservoir for zoonotic pathogens [2, 3]. Among arthropods, ticks have pivotal role as vectors for many zoonotic pathogens. A large proportion of tick-borne pathogens are bacterial. Nevertheless, several tick-borne or proposed tick-borne bacterial pathogens are not comprehensively studied and their geographical distribution is  insufficiently known.

For example, the spotted-fever group rickettsiae are a diverse group of alpha-proteobacteria, exclusively associated with arthropods, which may act as vectors or reservoirs in the life-cycles of these bacteria [4]. *Rickettsia* may cause febrile disease in humans, and some species also in dogs, with symptoms that range from mild to life-threatening [5]. More than 20 species with pathogenic potential for humans are described, and further species and subspecies have been proposed [6]. *Rickettsia* spp. are transmitted by ticks both transovarially, from the female tick to her offspring, without the need of a vertebrate reservoir, and transstadially, from a previous life stage, thereby requiring a vertebrate host to infect the previous life stage [4]. Consequently, the occurrence of transovarial transmission results in tick-borne pathogens that can be maintained in the tick population even in the (short-term) absence of vertebrate reservoirs. Also, other species within the alpha-proteobacteria are zoonotic pathogens of importance. The family *Anaplasmataceae* contains several species of zoonotic concern. For example, *Anaplasma phagocytophilum* infects humans and dogs, as well as a wide range of other domestic and wild animals [7, 8] and is known from Romania [9–11]. Other *Anaplasmataceae* species, such as *Ehrlicia cani*s, *A. platys* and “*Candidatus* Neoehrlichia mikurensis”, are in a few cases reported to occur in Romania [12–14], but the geographical distribution of these pathogens are not well known. Furthermore, several *Bartonella* spp. are pathogens in domestic animals that cause emerging diseases in humans [3, 15, 16], the distribution of *Bartonella* spp. are not known in Romania. Other neglected bacteria of interest from a zoonotic tick-borne disease perspective include *Francisella tularensis* and *Coxiella* spp. In order to further advance the knowledge regarding bacterial pathogens with zoonotic potential in Romania, we used molecular methods to screen ticks collected from animal hosts for the following pathogens: *Rickettsia* spp., *Bartonella* spp., *Anaplasma* spp., *Ehrlichia* spp., “*Ca.* Neoehrlichia mikurensis”, *Francisella* spp. and *Coxiella* spp.

## Methods

### Tick collection and identification

**Fig. 1:**
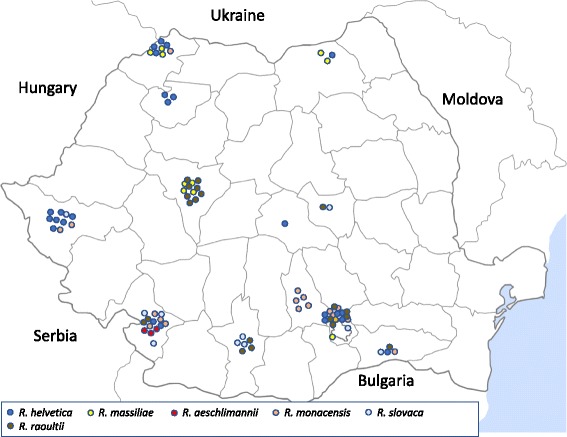
Geographical distribution of *Rickettsia* spp. in ticks collected from animals in Romania

Ticks were sampled from March 2014 to August 2015 in the following counties in Romania: Alba, Ilfov, Calarasi, Covasna, Dolj, Giurgiu, Gorj, Dambovita, Braila, Brasov, Mehedinti, Olt, Prahova, Timis, Salaj, Satu Mare, Sibiu, Suceava and Vilcea (Fig. 1). Ticks were collected mostly from pet dogs (*n* = 545) and few kennel or stray dogs, but also from other animals such as foxes from the rabies vaccination control program (*n* = 56), cats (*n* = 23), sheep (*n* = 75), rabbits (*n* = 5), cattle (*n* = 36), goats (*n* = 50), horses (*n* = 2) and turkey (*n* = 1). Additional questing ticks were collected from vegetation (*n* = 31) by flagging. Collected ticks were kept alive or preserved in ethanol and were identified to the taxonomic level of species using phenotypic keys [17, 18]. Some specimens were also genetically characterized as described in [19].

### Nucleic acid extraction and PCR

Ticks were homogenized using 200 μl Bio101 tissue lyser. Total nucleic acid was extracted using the MagNA Pure LC RNA/DNA Kit (Roche, Mannheim, Germany) in a MagNA Pure LC instrument (Roche, Mannheim, Germany) according to the manufacturer’s instructions. The total nucleic acid was stored at -80 °C until use. Most ticks were processed individually. However, a smaller number of nymphs were pooled in groups of ten (eight pools) and three (three pools).

Detection of *Rickettsia* spp. was performed using a previously published real-time PCR assay targeting a part of the *gltA* gene [20]. For species identification of *Rickettsia* an assay targeting the *23S* intergenic spacer region was utilized, and the amplicons were sequenced, as previously described [21]. Detection of *Anaplasma* spp., *Ehrlichia* spp. and “*Candidatus* Neoehrlichia mikurensis” was performed with a real-time PCR assay that amplifies a part of the *16S* rRNA gene as described [22]. All positive amplicons obtained with this assay were purified and sequenced, using the forward primer, as described in Andersson et al. [23]. Obtained sequences were compared with published GenBank sequences using Basic Local Alignment Search Tool (BLAST) analysis. Amplification of *Bartonella* spp. was performed using a real-time PCR assay targeting a region of the *ssrA*-gene with primers and time/temperature profile according to Diaz et al. [24]. The DNA extracts of all ticks were further screened for the presence of *Francisella* spp. DNA, using a real-time PCR assay targeting the *tul4* gene as described previously [25]. Finally, all samples were tested for the occurrence of *Coxiella burnetii* and *Coxiella*-like organisms using real-time PCRs for the target genes *IS1111*, *icd* and *com1* as described previously [26, 27]. Details regarding target species, amplified DNA fragment, positive control used in PCR and references are given in Table 1.

**Table 1:** Details regarding target species, amplified DNA fragment, positive control used in PCR and references

Target species	Target sequence	Primer sequences (5'-3'); forward, reverse and, where applicable, probe	Positive control	Reference
*Rickettsia* spp.	*gltA*	for: ATAGGACAACCGTTTATTT; rev: CAAACATCATATGCAGAAA; probe: FAM-CCTGATAATTCGTTAGATTTTACCG-TMR	DNA extract from positive cell culture of *R. monacensis* (own isolate)	[20]
*Anaplasma* spp.; *Ehrlichia* spp.; “*Ca.* N. mikurensis”	*16S* rRNA	for: GGGGATGATGTCAARTCAGCAY; rev: CACCAGCTTCGAGTTAAGCCAAT	DNA-extract from an *A. phagocytophilum-*positive *I. ricinus*	[22]
*Bartonella* spp.	*ssrA*	for: CTATGGTAATAAATGGACAATGAAATAA; rev: GCTTCTGTTGCCAGGTG	DNA-extract from *B. grahamii-* positive rodent blood	[24]
*Francisella* spp.	*tul4*	for: ATTACAATGGCAGGCTCCAGA; rev: TGCCCAAGTTTTATCGTTCTTCT; probe: FAM-TTCTAAGTGCCATGATACAAGCTTCCCAATTACTAAG-BBQ	*Francisella tularensis holarctica* Live Vaccine Strain (LVS = ATCC 29684	[25]
*Coxiella* spp.	*IS111*, *icd*, *com1*	IS1-for: CB_A2k: TCACATTGCCGCGTTTACT; IS1-rev: CBA_2k: TCACATTGCCGCGTTTACT; IS1-probe: Red640-TAATCACCAATCGCTTCGTCCCGGT; icdtrg_f: CGGAGTTAACCGGAGTATCCA; icdtrg_r: CCGTGAATTTCATGATGTTACCTTT; comtrg_f: CCCTGCAATTGGAACGAAG; comtrg_r: GTTCTGATAATTGGCCGTCGACA	DNA extract from cell cultured reference strain NineMile RSA 493	[26, 27]

*Abbreviations*: *for* forward, *rev* reverse

## Results

**Fig. 2:**
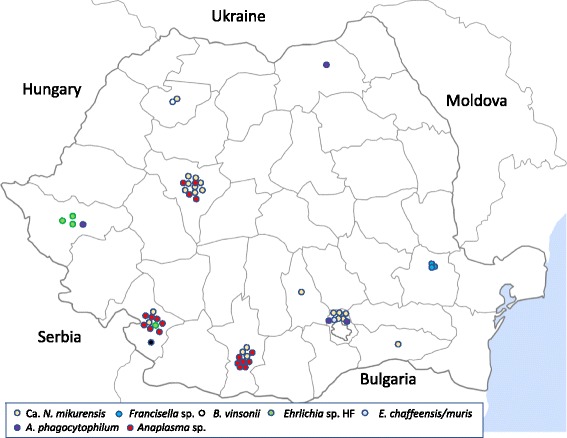
Geographical distribution of “*Candidatus* Neoehrlichia mikurensis”, *Francisella* sp., *Bartonella vinsonii berkhoffii*, *Ehrlichia* sp. HF, *Ehrlichia chaffeensis/muris*, *Anaplasma phagocytophilum* and *Anaplasma* sp. species in ticks collected from animals in Romania

In total, 824 ticks from 19 different counties were collected and screened for the presence of bacterial pathogens. Eleven tick species were identified: *Ixodes ricinus* (*n* = 209: 21 nymphs, 39 males, 149 females); *Ixodes crenulatus* (*n* = 7: 3 nymphs, 4 females); *Dermacentor marginatus* (*n* = 92: 7 nymphs, 30 males, 55 females); *Dermacentor reticulatus* (*n* = 119: 45 males, 74 females); *Haemaphysalis punctata* (*n* = 38: 29 nymphs, 9 females); *Haemaphysalis concinna* (*n* = 3: 1 nymph, 2 females); *Rhipicephalus sanguineus* “southeastern lineage” (*n* = 314: 143 nymphs, 64 males, 107 females); *Rhipicephalus rossicus* (*n* = 1 female); *Rhipicephalus bursa* (*n* = 15: 2 nymphs, 3 males, 10 females); *Hyalomma marginatum* (*n* = 26: 17 males, 9 females) and one *Hyalomma scupense*. Details about tick species and occurrence of respective pathogens are presented in Table 1. Males and females and nymphs were found to carry infections but none of the 11 pooled nymphs (eight pools with 10 nymphs/pool and three pools with 3 nymphs/pool) were positive for any pathogen. Geographical distributions of detected bacteria are shown in Figs. 1 and 2, Table 2, and Additional file 1: Table S1.

**Table 2:** Tick-borne bacteria in Romanian ticks; details regarding host species, tick species, life stage of ticks and area for each pathogen

Pathogen	Host	Tick species	Tick stage	Area
*Bartonella vinsonii berkhoffii* (*n* = 1)	dog	*Dermacentor reticulatus*	female	Zalau
*Rickettsia helvetica* (*n* = 24)	dog, fox, sheep, cat	*Dermacentor reticulatus, Ixodes ricinus*, *Rhipicephalus sanguineus*^a^, *Ixodes crenulatus*	female, male, nymph	Satu Mare, Brasov, Zalau, Calarasi, Corbeanca, Timisoara, Suceava, Jirov
*Rickettsia raoultii* (*n* = 21)	dog, sheep, goat	*Dermacentor reticulatus*, *Rhipicephalus sanguineus*, *Dermacentor marginatus*	female, male, nymph	Snagov, Calarasi, Sfantul Gheorghe, Targoviste, Livezile, Snagov, Jirov, Slatioara
*Rickettsia massiliae* (*n* = 10)	dog, fox	*Dermacentor reticulatus*	female, male	Satu Mare, Popesti Leordeni, Alba Iulia, Suceava
*Rickettsia monacensis* (*n* = 18)	dog, fox, cat	*Dermacentor reticulatus*, *Rhipicephalus sanguineus*, *Ixodes ricinus*	female, male, nymph	Satu Mare, Calarasi, Corbeanca, Timisoara, Targoviste, Picior de Munte, Jirov, Comanda
*Rickettsia slovaca* (*n* = 10)	dog, fox, sheep, goat, field	*Rhipicephalus sanguineus*, *Ixodes ricinus*, *Dermacentor marginatus*	female, male, nymph	Calarasi, Corbeanca, Sfantul Gheorghe, Timisoara, Jirov, Slatioara, Starmina
*Rickettsia aeschlimannii* (*n* = 3)	cattle	*Hyalomma marginatum*	female, male	Livezile
“*Ca.* Neoehrlichia mikurensis” (*n* = 1)	dog	*Ixodes ricinus*	female	Zalau
*Anaplasma phagocytophilum* (*n* = 4)	fox, dog, sheep	*Ixodes ricinus*	female, male, nymph	Corbeanca, Timisoara, Suceava
*Anaplasma marginale/ovis* (*n* = 16)	cattle, sheep, dog, goat	*Rhipicephalus bursa, Dermacentor marginatus*, *Ixodes ricinus*, *Dermacentor reticulatus*	female, male	Livezile, Ciochiuta, Jirov, Slatioara
*Ehrlichia chaffeensis/muris* (*n* = 1)	fox	*Ixodes ricinus*	female	Corbeanca
*Ehrlichia* sp. HF (*n* = 4)	dog	*Ixodes ricinus*, *Rhipicephalus sanguineus*	female, male	Timisoara, Jirov
*Francisella* spp. (*n* = 3)	dog	*Rhipicephalus sanguineus*	female	Braila

^a^*Rhipicephalus sanguineus* “southeastern lineage”

### *Rickettsia* spp.

**Fig. 3:**
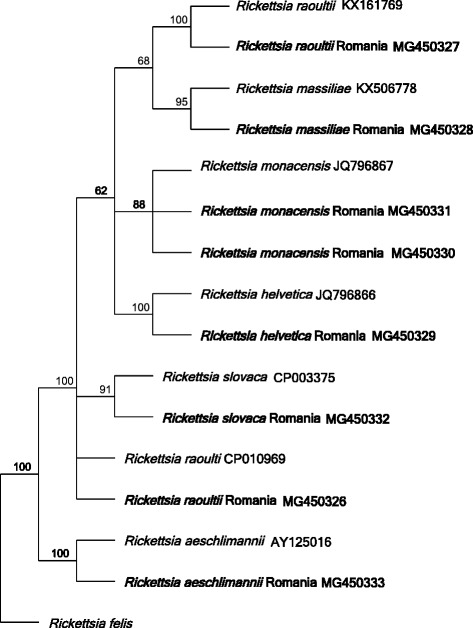
Phylogenetic relationships between *Rickettsia* species. Neighbor-joining tree with Jukes-Cantor as genetic distance model. Sequences obtained in the current study are in bold. The tree is based on 249 nucleotide positions of the *23S-5S* ribosomal RNA gene

In total, 10.6% (87/824) of the tick samples were positive, by means of real-time PCR, for *Rickettsia* spp. Positive samples were assigned to species level by sequencing of the *23S* ribosomal gene. The phylogenetic relationships for the obtained *Rickettsia* species are depicted in Fig. 3. The most common *Rickettsia* spp. was *R. raoultii*, which was detected in 2.5% (21/824) of the samples. Two different genotypes occurred, that differ by a 60 bp insertion/deletion. The most common genotype occurred in 14/21 samples (published in GenBank with the accession number MG450326). The sequence from this genotype did not completely match any previously published sequence in GenBank. However, it was highly similar (99.7% homology, 339/340 bp) to *R. raoultii* strain Khabarovsk (GenBank: CP010969), and similar (99.1% homology) to *R*. *raoultii* strain IM16 (GenBank: CP019435). Another *R. raoultii* genotype, with 100% homology to *R. raoultii* samples from *I. ricinus* in Austria (GenBank: KX161769) was detected in seven samples.

The sequences from ten samples showed 100% homology (339/339 bp) to *Rickettsia massiliae* isolate G266 (GenBank: KX506778). In 24 samples, sequences showing 100% homology to published *R. helvetica* sequences (GenBank: JQ796866 and EU057990) occurred (486/486 bp), collected from *I. ricinus* in Poland and Austria, respectively. *Rickettsia monacensis* was detected in 18 samples. Two different *R*. *monacensis* genotypes occurred. The most common genotype occurred in 17 samples, and the sequences were identical to each other and showed 100% similarity to *R. monacensis* isolate SzPK2-09 (GenBank: JQ796867). A second genotype was present in a single sample, and differed at two nucleotide positions. This sequence did not match any previously published sequence in GenBank. The closest match was the *R. monacensis* strain IrR/Munich, (GenBank: LN794217; 99.4% pairwise identity, 339/341 bp). Furthermore, 10 sequences were identical (341/341 bp) to several published *R. slovaca* sequences, for example *R. slovaca* strain D-CWPP (GenBank: CP003375). Finally, three *23S* sequences were identical (335/335 bp) to *R. aeschlimannii* strain MC16 (GenBank: AY125016). From a single sample that was positive with the *Rickettsia* spp. real-time PCR we did not manage to obtain any readable sequences and the species could therefore not be determined. The *Rickettsia* spp. sequences obtained in this study have been deposited in GenBank with the following accession numbers: *R. raoultii* (MG450326 and MG450327); *R. massiliae* (MG450328); *R. helvetica* (MG450329); *R. monacensis* (MG450330 and MG450331); *R. slovaca* (MG450332); and *R. aeschlimannii* (MG450333).

### *Bartonella* spp.

A single *I. ricinus* tick collected from a dog in Zalau, Salaj County, north-western Romania was positive for *Bartonella* spp. by means of real-time PCR. Sequencing of the amplicon demonstrated 100% homology (225/225 bp) to *B. vinsonii berkhoffii* str. Winnie. The obtained sequence has been deposited in GenBank with the accession number MG432827.

### *Anaplasma* spp., *Ehrlichia* spp. and “*Ca.* N. mikurensis”

**Fig. 4:**
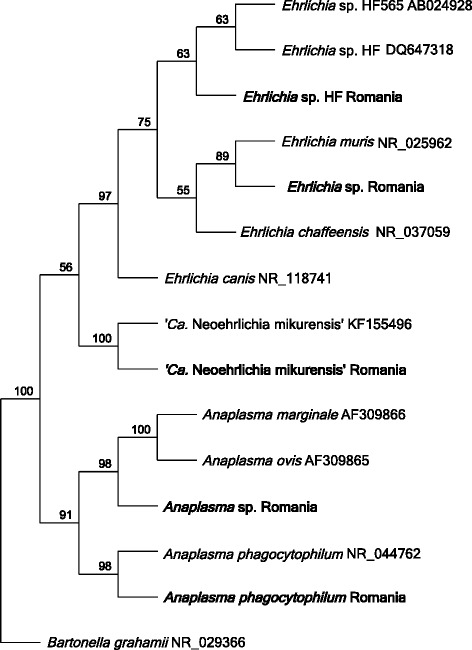
Phylogenetic relationship between *Anaplasma*, *Ehrlichia* and “*Ca.* Neoehrlichia mikurensis” species. Neighbor-joining tree with Jukes-Cantor as genetic distance model. Sequences obtained in the current study are in bold. The tree is based on 139 nucleotide positions of the *16S* ribosomal RNA gene

The amplicons of all samples positive with the *16S* rRNA assay were successfully sequenced. The phylogenetic relationship for the sequences obtained is depicted in Fig. 4. In four cases, the obtained sequences revealed 100% homology to published *Anaplasma phagocytophilum* sequences, for example the Webster strain with GenBank accession number NR_044762.1 (188/188 bp). All four samples were from *I. ricinus* collected from two foxes, a dog and a sheep. Sixteen sequences showed 100% homology to published *A. marginale* and *A. ovis* sequences, while differing from published *A. centrale* sequences at two nucleotide positions. The sequences from four samples detected in three *I. ricinus* and one *Rh. sanguineus* “southeastern lineage”, all obtained from dogs, showed 100% pairwise similarity (187/187 bp) to *Ehrlichia* sp. HF obtained from *I. ricinus* in Brittany, France (GenBank: DQ647318), as well as *Ehrlichia* sp. HF obtained from *I. ovatus* collected in Japan (GenBank: AB024928). An identical sequence has been reported from *I. apronophorus* in Romania (GenBank: KY851781). One sequence obtained from an *I. ricinus* collected from a dog in Zalau, Salaj County showed 100% pairwise similarity (171/171 bp) to published sequences of “*Candidatus* N. mikurensis” from various countries, for example sequences AB196305 and EU810404 from Japan and Germany, respectively.

A single organism closely related to *E. chaffeensis* was detected in an *I. ricinus* tick collected from a fox. This sequence was identical to the *E. chaffeensis* reference sequence NR_037059.1 (186/186 bp), however also to the *E. muris* reference sequence NR_121714.1, while differing from the *E. canis 16S* rRNA reference sequence NR_118741.1 at three nucleotide positions.

### *Francisella* spp. and *Coxiella* spp.

Three *Rh. sanguineus* “southeastern lineage” females, collected from dogs of a pound in Braila, were positive for *Francisella* spp. by means of real-time PCR, but due to the low amount of DNA it could not be further investigated by sequencing. All tested specimens were negative for *Coxiella* spp. by PCR.

## Discussion

We describe an abundant diversity of bacterial tick-borne pathogens in Romania. Previously, unidentified species, such as *Bartonella vinsonii berkhoffii* and *Francisella* spp., were detected as well as a diversity of genotypes within *Rickettsiaceae* and several *Anaplasmataceae* species. The most commonly detected pathogen in the current study was *Rickettsia* spp., occurring in over 10% of the ticks, distributed across the country and in several tick species. The following species were detected: *R. raoultii*, *R. slovaca*, *R. helvetica*, *R. monacensis*, *R. massiliae* and *R. aeschlimannii*, as well as different genotypes within some of these species. Rickettsiae are exclusively associated with arthropod vectors. For example, it is known that *D. marginatus* ticks are the main vector of *R. slovaca* and that *I*. *ricinus* ticks are the main vector of *R. helvetica* [5, 28, 29]. *Rickettsia slovaca* has also been detected in *D*. *reticulatus* ticks [5, 30]. In Europe, there are numerous reports on the prevalence of zoonotic rickettsiae in *D. reticulatus*, mainly on the causative agents of tick-borne lymphadenopathy (TIBOLA), e.g. *R. raoultii* and *R. slovaca* [31–33]. *Dermacentor reticulatus* is also known as a carrier of *R. raoultii* and *R. slovaca* [34], but also *R. helvetica* [31]. In Croatia, *R*. *conorii*, *R*. *slovaca, R. helvetica* and *R*. *aeschlimannii* have been detected in *Rh. sanguineus*, *D. marginatus* and *Hyalomma marginatum*, respectively [31, 35].

In the present study, no *R. conorii* was detected. Nevertheless, the data discussed by Serban et al. [36] suggest a sporadic human infection in Constanta county and the authors conclusion that the distribution of cases matches with the distribution of *R. conorii* and its tick vectors, and also with the period of greatest activity of the vector *Rh. sanguineus*, is not supported by the scientific data. Ionita et al. [37] reported one case of *R. conorii*, but without giving details about the place where the tick was collected. The same authors detected other *Rickettsia* spp. in ticks from Romania, e.g. *R. raoultii* (16%) and *R. slovaca* (3%) in *D. reticulatus*, and *R. monacensis* (11%) in *I. ricinus* [37]. Marcutan et al. [38] reported the first individual records of different *Rickettsia* spp. in *H. concinna* (*R. monacensis*), *I. arboricola* (*R. helvetica*, *R. massiliae*) and *I. redikorzevi* (*R. helvetica*) and also the first geographical record on the occurrence of *R. massiliae* in Romania, representing the easternmost observation in Europe. These results confirm earlier work on the geographical distribution of *Rickettsia* spp. in Romania, and increase our knowledge regarding the geographical distribution of these species. As most of the *Rickettsia* spp. identified and described here are known to cause human disease they should be considered in differential diagnosis of febrile or exanthematous clinical disease in Romanians, and in travelers from Romania.

The bacterial genus *Bartonella* contains several species of zoonotic concern. *Bartonella vinsonii berkhoffii* was first reported in a dog with endocarditis [16] and have been reported to cause human infection with arthralgia, fatigue and neurological symptoms in immunocompetent individuals [15]. Several strains of this subspecies have been described [39]. The strain Winnie, with a *ssrA* sequence identical to the one obtained from Romania in the current study, was originally isolated from a dog in USA [3]. In Romania, a large proportion of human patients diagnosed with haematologic cancer and undergoing chemotherapeutic treatment were shown to be seroreactive against *Bartonella* spp. antigens. This was especially common in patients living on farms with animal contact [40].

The presence of *Ehrlichia* sp. HF in four samples, all collected from dogs, is noteworthy. In Romania, this bacterium has previously been detected in *I. apronophorus* collected from dogs and a fox [41]. Prior to this report, *Ehrlichia* sp. HF had not been reported from Romania. Nevertheless, in the present study it was the most commonly detected *Ehrlichia* species. Nothing is known, so far, either on the transmission cycle or on the pathogenesis of this *Ehrlichia* species. The repeated detection, however, warrants further studies on this species potential role as a pathogen in dogs and perhaps humans.

A single case of an *Ehrlichia* sp. closely related to *E. chaffeensis* occurred in a tick collected from a fox. It is not possible to distinguish *E. chaffeensis* and *E. muris* based on the sequence fragment obtained in the current study. Nevertheless, the related canine pathogen *E. canis* differs by three nucleotides and can thus be readily differentiated. The occurrence of this *Ehrlichia* sp. requires further investigation with molecular methods in Romania.

“*Candidatus* N. mikurensis” was detected in an *I. ricinus* collected from a dog in north-eastern Romania. This species has been reported in *I. ricinus* from several counties in the western and central part of Romania [13, 14] and it seems widespread in the country. Infection with “*Ca.* N. mikurensis” in a dog with chronic neutropenia has been reported in a single case from Germany [42]. A study investigating 96 dogs with suspected tick-borne infections from southern Romania did not show any case of infection with “*Ca.* N. mikurensis” [43]. The degree to which “*Ca.* N. mikurensis” can cause infection in dogs remains unclear. The relatively high prevalence in *I. ricinus* [44], the geographical widespread occurrence and the frequent exposure of dogs to questing ticks does nevertheless suggest that more studies need to be conducted on the subject.

The closely related *Anaplasma* species (*A. marginale* and *A*. *ovis*) could not be readily differentiated based on the partial *16S* rRNA fragment amplified in this study. The two species are closely related and differs at just a few positions in the complete *16S* rRNA gene. For example, the *A. marginale* strain ‘Virginia’, isolated from a cow in southern Virginia and *A*. *ovis* strain ‘Idaho’ isolated from a sheep, are 99.7% similar in the *16S* rRNA gene.

Three *Rh. sanguineus* “southeastern lineage” females were positive for *Francisella* spp. As the ticks were collected from dogs, it is possible that the ticks were infested with the bacterium either as a larva or nymph during previous feeding or during feeding as adults on the dog. Regardless of when the ticks were infected, this is the first detection of *Francisella* spp. in *Rh. sanguineus* “southeastern lineage” in Braila, Romania. Due to the low amount of DNA the species of *Francisella* was not identified. *Rhipicephalus sanguineus* and *Rh. rossicus* are known as wetland ticks in Romania, and especially *Rh. rossicus* might have a role as a vector in the transmission of *F. tularensis* [45, 46]. Another tick species, *Ixodes apronophorus*, is considered as a potential vector of *F. tularensis* [47]. All ticks positive for *Francisella* spp. in the present study were from the same dog pound. Tularemia may cause clinical symptoms in dogs, and the health consequences in the local dog pound could preferentially be further studied. Moreover, dogs may act as carrier of the pathogen across countries.

Nevertheless, *Francisella*-like endosymbionts of ticks are known and such organisms have been reported in various tick species from different countries: *Haemaphysalis flava* and *H. phasiana* in the Republic of Korea [48], *Dermacentor reticulatus* in Portugal [49], Bulgaria [50] and Hungary [51], *Rh. sanguineus* (*s.l.*) in Bulgaria [50] and Thailand [52], and in *Hyalomma* in Israel [53] and Bulgaria [50]. It was not detected in ticks investigated in Turkey [54].

## Conclusions

A substantial diversity of bacterial tick-borne pathogens was observed when screening ticks collected from animal hosts in Romania. This diversity was especially evident by the number of different species and genotypes of *Rickettsia* detected. Some of the findings in this study are novel for Romania, such as the detection of the zoonotic *Bartonella vinsonii berkhoffii* in a tick collected from a dog. Three cases of *Francisella* spp. were found in *Rh. sanguineus* collected from dogs. Taken together the results presented in this study warrant further studies on the consequences of tick-borne pathogens in domestic animals in Romania.

## Additional file

Additional file 1: **Table S1.** Tick-borne bacteria in Romanian ticks; details regarding tick species, locality, county, year, host, life stage of ticks and pathogen. (XLS 17 kb)
